# Reiki as a tool to enhance care from the perspective of complexity: a qualitative study

**DOI:** 10.1590/0034-7167-2025-0082

**Published:** 2025-11-07

**Authors:** Silvana Possani Medeiros, Adriane Maria Netto de Oliveira, Mara Regina Santos da Silva, Andreia Martins do Couto, Vagner Ferreira do Nascimento, Silomar Ilha

**Affiliations:** I Universidade Federal do Rio Grande. Rio Grande, Rio Grande do Sul, Brazil; II Espaço Terapêutico Andréia Martins do Couto. Rio Grande, Rio Grande do Sul, Brazil; III Universidade do Estado de Mato Grosso. Cáceres, Mato Grosso, Brazil; IV Universidade Federal de Santa Maria. Palmeira das Missões, Rio Grane do Sul, Brazil

**Keywords:** Therapeutic Touch, Nonlinear Dynamics, Qualitative Research, Nursing Care, Nursing., Tacto Terapéutico, Dinámicas no Lineales, Investigación Cualitativa, Atención de Enfermería, Enfermería.

## Abstract

**Objectives:**

to describe the contributions of Reiki as a tool to enhance care.

**Methods:**

exploratory, descriptive, qualitative study grounded in the Complexity framework. Ten nurses from a 24-hour Primary Health Care Unit in southern Brazil participated. Data were collected between April and August 2022 through individual semi-structured interviews after each participant received five Reiki sessions. Thematic content analysis was used.

**Results:**

Reiki promoted changes in nurses’ feelings, self-knowledge, and self-awareness, which were reflected in altered behaviors and attitudes when facing daily uncertainties. Participants reported new ways of (co)existing and engaging with order, disorder, and reorganization processes. These changes contributed to healthier eating, improved sleep quality, greater disposition and energy for daily tasks, and the adoption of regular physical activity.

**Conclusions:**

Reiki proved to be a tool that enhances complex care and supports health and nursing professionals in coping with the disorders experienced in daily life.

## RESUMEN

**Objetivos:**

describir los aportes del Reiki como herramienta para potenciar el cuidado.

**Métodos:**

estudio exploratorio, descriptivo y cualitativo, basado en el marco teórico-conceptual de la Complejidad. Participaron 10 enfermeros de una Unidad Básica de Salud con atención de 24 horas, en el sur de Brasil. Los datos se recolectaron entre abril y agosto de 2022 mediante entrevistas semiestructuradas individuales, realizadas tras cinco sesiones de Reiki. Se aplicó análisis de contenido temático.

**Resultados:**

el Reiki promovió cambios en los sentimientos, el autoconocimiento y la autoconciencia de los enfermeros, reflejados en nuevas actitudes frente a las incertidumbres cotidianas y en formas distintas de (co)existir y afrontar procesos de orden-desorden-reorganización. Estos cambios incluyeron mejoras en alimentación, sueño, disposición para actividades diarias y práctica de actividad física.

**Conclusiones:**

el Reiki se mostró como una herramienta que potencia el cuidado complejo y ayuda a los profesionales de salud/enfermería a afrontar trastornos de la vida diaria.

## INTRODUCTION

Several factors affect humankind, including how individuals interpret and cope with problems that arise throughout life. Stress, a psychophysiological response to intrinsic and extrinsic agents, stands out among these factors. It can generate disorders that manifest through biopsychosocial dysfunctions, such as increased heart rate (HR) and systemic blood pressure (SBP), sweating, myalgia, anxiety, fatigue, irritability, insomnia, eating disorders, decreased ability to concentrate, and other harmful effects on human health. The intensification of stimuli reduces an individual’s overall performance in daily life and well-being^(1)^. Various changes may occur when stress is not properly monitored and treated^(2)^.

Although stress can occur anywhere and at any time in life, the workplace is a setting that can foster it, depending on the nature of the work. In this context, the work of healthcare professionals especially nurses stands out, as their clinical and managerial roles take place in environments filled with elements and conditions that may trigger stress-related disorders. Thus, nurses must continuously (re)organize their behavior in response to stressors to maintain their biopsychosocial and spiritual balance. These agents may originate from internal and/or external sources, such as emotions arising from daily professional experiences or exposure to unhealthy work environments^(3)^.

In this context, strategies must be (re)constructed and implemented in the work environment. Thus, the importance of Complexity Thinking is highlighted, as it emphasizes the need to move beyond isolation, disconnection, or juxtaposition, proposing instead a unified, connected, attentive, and comprehensive mode of thought^(4)^. Such an approach enables the promotion of complex care for healthcare professionals, where one embraces certainty and uncertainty, the separable and the inseparable, without simplifying or reducing one’s lived experiences.

From the perspective of Complexity Thinking, nursing care practices must consider the circularity of propositional situations of order, disorder, and organization that emerge in workplace interactions. A study conducted in Bahia, Brazil, describes that complex care in nursing must encompass the multidimensionality of the human being, taking into account not only physical aspects but also emotional, social, and spiritual dimensions^(5)^. This understanding must apply when care is directed toward the individuals served by professionals and the professionals themselves as recipients of care.

Thus, the concept of complex care adopted in this study refers to care encompassing the multiple dimensions of human beings whether patients or professionals that interact to form a whole. This process is shaped by how these dimensions relate to each other. Therefore, in the context of nursing professionals and their work environment, strategies are needed to promote harmony and balance among the various dimensions that constitute them.

Among the possible strategies to promote the care of healthcare professionals, Reiki stands out as a therapeutic practice. Originating from Japanese culture, it involves the laying on of hands to channel vital energy and stimulate the body’s natural mechanisms of recovery and health maintenance^(6)^. This practice is employed to provide complex care, in which the Reiki therapist serves as a channel for the flow of universal energy to others, acting on different levels and harmoniously balancing the Chakras energy centers associated with the body’s main glands to reestablish homeostasis. Through the Chakras, the human body connects with, absorbs, and transforms the energy that permeates it, distributing it throughout the body and contributing to health processes^(7)^.

In this sense, Reiki is an Integrative and Complementary Health Practice (ICHP) that addresses multidimensional biopsychosocial-spiritual care, considering the human being as unique and integrated. These characteristics inherently define it as a phenomenon that promotes complex care. However, further research is needed to reaffirm the contributions of Reiki as a practice that enhances complex care. Although studies have explored the use of Reiki as a therapeutic practice in various contexts^(8-11)^, this study proposes a discussion grounded in the theoretical framework of Complexity Thinking more specifically, in the concepts of order, disorder, and (re)organization^(4)^ which underscores the relevance of this research.

Furthermore, this topic is linked to the third objective “health and well-being” for sustainable development, as outlined in the 2030 Agenda of the United Nations (UN^)(12)^. It also aligns with the recommendations of the document “World Health Organization (WHO) Traditional Medicine Strategy: 2014-2023”, which guides and encourages the use of traditional practices as broad complementary treatments within health systems^(13)^. From this perspective, the guiding question is: What are the contributions of Reiki as a tool to enhance care?

## OBJECTIVES

To describe the contributions of Reiki as a tool to enhance care.

## METHODS

### Ethical aspects

This study derived from a larger project titled “*Promoção da saúde mental ao longo do desenvolvimento humano*” [Promotion of mental health throughout human development], approved by the Institutional Review Board of the Federal University of Rio Grande. The study followed the recommendations of Resolution 466/12^(14)^, which establishes guidelines and regulations for research involving human subjects. Participants signed an informed consent form in two copies (one for the participant and one for the researchers). They are identified by the letter “N” (nurse) followed by a number indicating the order of the interviews (e.g., N1, N2, up to N10), ensuring the confidentiality of their identities.

### Study design

This exploratory, descriptive, and qualitative study complied with methodological rigor criteria for qualitative research and followed the Consolidated Criteria for Reporting Qualitative Research (COREQ)^(15)^.

### Study setting

A survey was conducted at a 24-hour Primary Health Care (PHC) unit located in the interior of Rio Grande do Sul, Brazil. In addition to serving the local community, this unit also provides urgent and emergency care and more complex interventions to residents of other neighborhoods. It also serves patients who cannot schedule appointments at their neighborhood PHC units^(16)^.

### Population

There were 36 healthcare professionals working in the setting during the study period: 10 nurses, 16 nursing technicians, nine physicians, and one dentist. Nurses who were present during the data collection period and met the selection criteria were included. These professionals were chosen due to their heavy workload, as nurses are responsible not only for providing direct patient care but also for managing the service.

### Participant selection criteria

The inclusion criterion was being a nurse working at the 24-hour PHC unit. The exclusion criterion was being on leave during the data collection period. All nurses assigned to the unit the research setting met the inclusion criteria and agreed to participate in the study, totaling 10 professionals.

### Data collection

Data were collected between April and August 2022 in six meetings with each participant. In the first meeting, the study was presented to the nurses, and they signed a consent form. From the second to the sixth meeting, individual Reiki sessions were held once a week with all participants. One of the researchers a nurse and Reiki therapist with three levels of qualification in the technique (Reiki levels I, II, and III) conducted the sessions. The sessions took place at the 24-hour PHC unit, in an office made available at the time. Scheduling was based on the participants’ availability.

Each Reiki session lasted 20 minutes and was conducted as follows: The room was prepared beforehand with relaxing music and an aroma diffuser to enhance comfort and create a harmonious environment. The participant was invited to lie on a stretcher in a supine position and was verbally encouraged by the researcher to close their eyes and visualize positive images and affirmations during the session to foster a deeper connection with the practice.

Next, the researcher took a moment to focus before beginning the Reiki application, positioning her hands palm-down in a shell-like shape. The session began with harmonizing the crown and third-eye chakras, corresponding to the head’s frontal, temporal, and occipital regions. This practice was followed by the laryngeal chakra, located in the supraand infra-hyoid regions; the heart chakra, in the sternal region; the solar plexus chakra, in the epigastric region; the sacral chakra, in the mesogastric region; and the root chakra, in the pubic region. While the participant remained supine, the secondary knee chakras (patellar region) and foot chakras (plantar region) were also accessed. The participant then turned to the prone position, allowing the researcher to work on the heart chakra in the scapular region and the solar plexus chakra in the dorsal region. The session concluded with harmonizing the sacral chakra in the lumbar region.

The sixth meeting was held one week after the last Reiki session with each participant. During this meeting, a previously trained researcher with experience in data collection techniques conducted individual interviews in the same office where the Reiki sessions had taken place. The interviews lasted an average of 30 minutes and were guided by a semi-structured script developed specifically for this study. The script consisted of two parts. The first part addressed participant characteristics such as gender, age, time since graduation, time working at the unit, income, medication use, alcohol consumption, physical activity, leisure habits, and religion. The second part included the following open-ended questions: Tell me how you felt before the Reiki sessions. Did Reiki influence your daily life? In what way? Did you notice any changes in your life after receiving Reiki? What changes?

The interviews were audio-recorded and manually transcribed verbatim by the researchers using Microsoft Word (version 16.31).

### Data processing and analysis

Data were analyzed using the thematic content analysis technique^(17)^, following these steps: 1) Pre-analysis, during which floating reading was conducted to skim the material; 2) Exploration of the material, involving an in-depth reading of the interview transcripts to identify statements that elucidated the contribution of Reiki as a tool to enhance complex care and any changes perceived in daily life after its application. At this stage, units of meaning were extracted and grouped into categories; 3) Treatment of the results and their interpretation, based on the Complexity framework^(4)^.

## RESULTS

**Figure 1:**
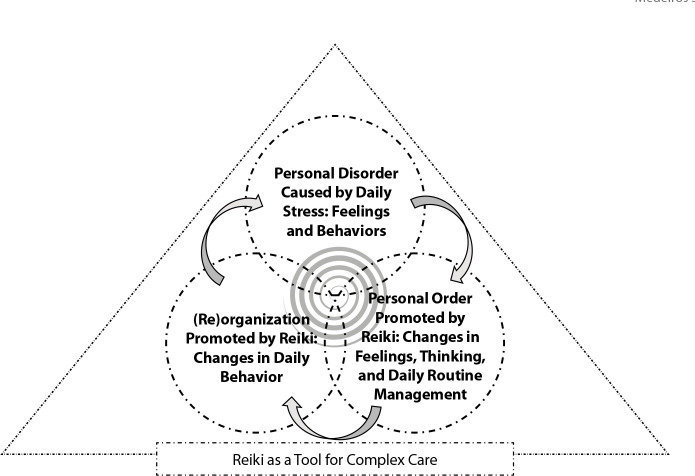
Schematic representation of categories based on the Complexity framework

Of the 10 nurses who participated in the study, seven were women, and three were men, with an average age of 35. They had an average of nine years of professional experience and an average of 1.8 years working at the PHC unit. Reported incomes ranged between three and four times the minimum wage. Five participants lived only with their children, while five lived with their partners. In terms of lifestyle, six reported consuming alcohol at least once a week, three used controlled medication (anxiolytics), and five engaged in physical activity. As for leisure activities, the most common was watching TV series and movies (n = 7). Five professionals reported having a religion, with Catholicism being the most prevalent (n = 3). Three categories emerged from the data (Figure 1).

### Personal Disorder Caused by Daily Stress: Feelings and Behaviors

The way individuals experience stress is unique, shaped by their beliefs and personal experiences. However, certain aspects were shared in the daily work of the nurses who participated in this study, particularly the sense of personal disorder caused by excessive stress. The accounts of two participants revealed the presence of daily stress combined with experiences of uncertainty specifically, the COVID-19 pandemic which led to mood swings, irritability, and impatience.

*Of course, I get stressed out. For example, today I’m a nervous wreck tense because, in addition to COVID-19, I have to make several requests, send reports, and, in short, handle all the administrative and management tasks of the unit so that they have the minimum material resources necessary. It’s all very stressful! It all makes me really angry!* (N1)

*I get offended easily, so whenever someone would say something to me, I’d argue. Sometimes I wasn’t even right, but even if I thought I wasn’t, I’d still get stressed out. Especially with COVID-19, we got more nervous with our nerves on edge.* (N4)

The feelings resulting from the disorders caused by work-related stress had repercussions not only on daily professional life but also on aspects of personal and family life.

*I was dealing with a lot of problems at home I was angry with my son, I’d argue a lot and even swear. And to make matters worse, my colleague on duty, the other nurse we both have strong personalities and we had some arguments that weren’t pleasant. I even thought about asking for a change of shift or duty day because working with him was very difficult and stressful.* (N7)

*I had no patience at all some people would make me sick just by coming near me. I felt like a burden. It was unbearable having to put up with some people, especially here at work, because there are some very difficult people not just colleagues, but also some patients.* (N10)

The nurses’ accounts reveal the disorder caused by stress and a lack of self-understanding particularly in terms of self-awareness and emotional clarity which, in some cases, made it difficult for them to recognize what was truly affecting their daily lives. In addition to changes in emotional states, work-related problems also influenced behaviors detrimental to their health, such as poor sleep quality, inadequate nutrition, and a lack of motivation to carry out work activities.

*I had a very restless sleep it took me a long time to fall asleep. There were days when it felt like I hadn’t even slept because I’d wake up more tired than when I went to bed* [...] *I noticed my anxiety and compulsive eating because of how much I was eating. I needed to eat all the time, and the food was really poor quality often only fast food would satisfy me.* (N3)

*My sleep has been kind of poor, you know? It feels like I’m always tired, like there’s heaviness in my body. It must be from the tension related to this disease COVID-19 that never ends. On top of that, the daily tasks here at work are tense, stressful. I leave exhausted.* (N5)

*I’ve been eating poorly, because I’m one of those people who takes their anxiety out on food. I’ve been eating a lot of sweets and fast food* [...] *usually, when I got home from work, I’d feel exhausted and had no desire to leave the house or see anyone just to sleep.* (N9)

The reports reveal the disorder resulting from stress in the nurses’ daily lives. The disorganization of their routines both in the family sphere and at the work environment generated feelings of irritability, nervousness, and fatigue, leading to careless behaviors.

### Personal Order Promoted by Reiki: Changes in Feelings, Thinking, and Daily Routine Management

The disorder manifested through feelings of discouragement, overload, impatience, and mood instability gradually began to be reinterpreted through Reiki, resulting in a new order that is, new ways of seeing, (co)existing, and managing daily activities. After the Reiki intervention, some nurses adopted a different posture, developed a new awareness of themselves, and began to handle situations that were previously unfamiliar to them, as shown in the following accounts:

*Now, after receiving Reiki, I’m thinking: Oh, I have to do this thing. But, if I can’t do it, I can’t do it I’m not going to torture myself and suffer because of it. You know? I’ve been breathing a lot more, trying to calm down. I’ve been reflecting more.* (N1)

*Ah, it’s like I’ve been able to deal better with the adversities of everyday life. I’ve had a bit more emotional balance to handle the problems we face at home and at work in life in general. I’m calmer after receiving Reiki.* (N2)

*I realize that now I think more about myself I breathe more slowly, think more clearly, and do things more calmly and peacefully, focusing on what’s really important to me. Thank you so much for Reiki; it really helped me.* (N8)

*Now, after receiving Reiki, I try to look at things from a more positive perspective. I try to filter what I’m hearing if someone close to me is talking, I focus on what’s important for me to hear, whether it will add something to my life or not. I’m changing that, and it helps me a lot.* (N10)

The participants’ accounts reveal a change in their thinking and, consequently, their behaviors when dealing with day-to-day uncertainties after the Reiki intervention. The nurses reported that a new order had been established based on the transformations they experienced and the dynamics they introduced into their routines. They began a restructuring process internally, through a new order of thought, and externally, through a new order of behavior.

### (Re)organization Promoted by Reiki: Changes in Daily Behavior

The nurses’ experiences demonstrate that, following the Reiki intervention, in addition to a new order brought about by changes in their ways of thinking and feeling, a process of (re)organization took place, reflected in altered behaviors. A cycle emerged in which disorder gave way to a new order, leading to a (re)organization of daily life.

*I’m feeling calmer after starting the Reiki sessions. I’ve calmed down a bit, and I’ve reorganized things in my life in general. I haven’t been irritated* [...] *I’ve tried to move around a bit, to do some physical activity I’ve started walking, not every day, but I already feel more energetic.* (N1)

*Now I’m allowing myself to do other things outside of work. Sometimes I go for a bike ride, sometimes I go for a walk, and I’m not always focused on the things that stress me out-on my problems. I think I’m organizing myself better now.* [...] *I’m even less anxious about eating eating less and in a more balanced way, eating fruits, which I didn’t eat before, eating more slowly, drinking more water because work will still be there when I get back from my break.* (N3)

*I used to feel pain in my feet from standing 12 hours here at the clinic, and pain in my lower back but after I did Reiki, it went away right away. Especially on the day we had the session, but overall it has improved I’ve remained pain-free* [...] *After Reiki, I sleep better, without interruptions, with better quality. I wake up less tired. This ends up giving me more energy during the day I have more energy and enthusiasm to do things.* (N4)

*I noticed that my relationship with my parents has improved, and they say I’ve become calmer that I’ve started speaking more calmly, with more patience, after the Reiki sessions* [...] *I feel like I have more energy now. I’ve even gone back to working out, doing weight training. I even have more energy to study.* (N6)

The nurses’ accounts reveal that it was possible for them to (re)organize themselves to manage daily stress through the use of Reiki as a complex care tool. Certain emotions and behaviors were (re)organized, giving rise to a new order marked by changes in the very elements that had previously led to disorder.

## DISCUSSION

The complexity of being a nurse encompasses various aspects related to care delivery in diverse situations, taking into account the complexity of the human being, the challenges of interpersonal and interprofessional relationships, the connection between caregiver and care recipient, the reality of life’s finitude, and the continuous deepening of knowledge regarding the complexity of care within the context of comprehensive care^(18)^. In this sense, nurses may experience stressful situations as they work directly in care delivery, often facing moments of uncertainty, fear, and feelings of helplessness in the face of suffering and death.

This study revealed the disorder caused by stress in the lives of nurses, which led to the disorganization of their routines and affected both their family life and work environment. This disorder was expressed through feelings such as irritability, nervousness, and fatigue, which were intensified by the COVID-19 pandemic. These findings are consistent with those of a literature review that examined the impact of the COVID-19 pandemic on nurses’ mental health. The study showed that healthcare professionals faced intense emotions and feelings in the workplace and experienced high levels of stress and tension, which increased the risk of irritability, apathy, discouragement, anguish, and anxiety, among other emotional states^(19)^.

In this regard, it is worth noting that public health emergencies such as the COVID-19 pandemic can lead to increased rates of anxiety, depression, and other mental disorders among healthcare professionals. They may also promote negative social behaviors such as irritability and impatience, which can directly impact their biopsychosocial health and the effectiveness of their professional performance^(20)^. In addition to emotional and psychological distress, exposure to high levels of stress led the nurses in this study to adopt unhealthy behaviors, such as inadequate diets, non-restorative sleep, and persistent fatigue upon waking, resulting in discouragement toward work. It is important to highlight that, in stressful situations, inappropriate eating behaviors emerge, often justified as a form of food reward. This includes eating without hunger and choosing less nutritious foods high in fat and carbohydrates. In this way, the disorder produced by stress contributes to disordered eating behaviors, often associated with binge eating^(21)^.

An international study conducted with nurses caring for patients with COVID-19 reported similar findings. It showed that professionals experienced high levels of stress that directly impacted the quality of their sleep and rest whether due to insufficient sleep duration, nightmares, frequent interruptions, or difficulty waking in the morning^(22)^.

This study shows that the participants perceived the emergence of a new order after the five Reiki sessions, as the nurses began to understand that setbacks and adversities would continue to occur in their daily lives and that it was impossible to control everything around them, given their constant exposure to unexpected situations. Some reports indicated a shift in how they dealt with adverse emotions and daily challenges, grounded in the realization that many of these situations were beyond their control. In terms of interpersonal relationships initially perceived as difficult, tense, and stressful the results suggest that participants became more accepting of differences. They recognized that, although some issues could not be changed, maintaining a calm mind was more beneficial, and change began to occur through reflection and the redefinition of attitudes and behaviors.

Changes in thinking occurred because Reiki encourages individuals to look inward, fostering self-knowledge and reflection on their surroundings while promoting a sense of security, well-being, and positive emotions^(7)^. Therefore, Reiki, as a care tool, facilitated an expansion of consciousness, leading participants to self-analysis and a deeper understanding of life. As a result, the nurses developed a new perspective on events, with greater discernment and improved ability to cope with everyday adversities.

In this sense, Reiki demonstrated its contribution as a tool that enhances complex care, as it promoted a new order of thought and feeling that enabled the (re)organization of behaviors related to food, fostering more balanced dietary choices. The findings of this study reaffirm the principles outlined in the Brazilian Policy on Integrative and Complementary Practices, which recognizes Reiki as a practice that promotes the integration of the psychic, emotional, physical, and spiritual dimensions, acknowledging the singularity and multidimensionality of the human being^(6)^. This perspective contrasts with the medical-centered model, which tends to fragment the human being into isolated parts without necessarily considering the whole.

The participants reported improved sleep quality, increased energy and willingness to carry out daily activities, and the resumption of regular physical activity following the Reiki sessions. These results are consistent with those of a study in which nurses experienced the positive effects of Reiki on sleep quality and reported a reduction or even elimination of physical pain^(23)^. Other studies examining the impact of Reiki on nurses’ daily lives reported similar findings, including improved sleep quality, reduced pain and daytime fatigue, greater motivation for daily activities^(24,25)^, increased self-esteem, and enhanced awareness of oneself, family, work, and life in general^(25)^.

These results were made possible because Reiki unblocks energy, allowing individuals to feel calmer through muscle relaxation and the production of serotonin and endorphins hormones that promote well-being and relaxation. As a result, the body becomes harmonized and balanced, which in turn promotes healthier food choices and increased hydration, enhancing the detoxifying effects of Reiki and supporting its benefits^(7)^. The increased sense of well-being and vitality arises from the intentional channeling of energy aimed at restoring balance to the energy field inherent to all living beings, which subsequently contributes to improved well-being across all dimensions^(7)^.

This positive outcome is expected, as Reiki is a tool for promoting the balance of the Chakras, which influences the endocrine glands, supports the homeostasis of the nervous system, dissipates emotional traumas, and releases stagnant emotions. It also promotes the expansion of consciousness, fosters a connection with Universal Energy, deepens the individual’s connection with their higher self, and enhances sensitivity, creativity, and intuition.

Thus, Reiki positively impacted the professionals’ daily lives, as evidenced by the (re)organization of various aspects of their routines. This finding is consistent with a study conducted in the United States, in which participants were asked to report their experiences during Reiki sessions by completing a questionnaire immediately after the sessions. The researchers collected 1,284 responses, which were grouped into eight main categories: increased relaxation and deep tranquility (68%), amplification of bodily sensations and positive somatic experiences (53%), improvement in emotional regulation and control (29%), spiritual or symbolic meaning (18%), improvement in physical symptoms related to pain (17%), reflective states and changes in perception (11%), induction of sleep and drowsiness (10%), and stabilization of breathing (4%)^(26)^.

Another study, conducted by the State University of Rio de Janeiro, aimed to reduce the stress burden of healthcare professionals through comprehensive care using Integrative and Complementary Health Practices (ICHP), such as auriculotherapy, Reiki, foot reflexology, and massage, and reported positive results. In this context, ICHP proved to be an effective and feasible strategy for supporting the care of healthcare workers. The incorporation of these practices into the work setting fostered the integration of knowledge, the connection of practices and experiences, and the promotion of mental health and quality of life through effective care interventions^(27)^.

It is important to note that ICHP do not aim to isolate or separate, but rather to promote coexistence between complementary rationalities and practices that offer more comprehensive care approaches. These practices contribute to the well-being of healthcare workers and, consequently, to the quality of care they provide^(27)^. In this sense, the meanings and experiences associated with Reiki are diverse. However, they converge in recognizing the practice as a source of health, well-being, and quality of life by providing multidimensional care to the human being^(28)^.

This perspective acknowledges the complexity of change processes and the interrelationships between different levels and dimensions of the same phenomenon. Within this paradigm, care delivery always involves another individual as the recipient, constituting a two-way interaction that requires the establishment of bonds to ensure the effectiveness of helping the other become the protagonist and promoter of their own health. Reiki, like other ICHP, aligns with this principle by naturally fostering the creation and strengthening of bonds, promoting multidimensional care in which the human being is considered in their singularity-as well as in the context of how their individuality interacts with others and various environments, all of which influence their health and quality of life^(29)^.

The Complexity framework emphasizes that human beings should not be confined to a single field of knowledge, as human unity is constituted by multidimensionality, and complex thinking highlights alternative ways of applying logic^(5)^. From this perspective, nursing care must be understood as a social and dynamic practice one that acknowledges biological, social, cultural, and political similarities and differences while also recognizing the complexity of the interrelationships among human beings and within the care process.

Therefore, Reiki, as a tool for complex care, emerges as a form of support for health and nursing professionals that also impacts the care they provide. It represents an innovative praxis with the potential for renewal, considering the full range of experiences available to consciousness. In this sense, Complexity and Reiki share a close relationship, as both integrate and connect diverse bodies of knowledge across various fields while preserving the essence and guiding thread of singularitiesthat is, reconnecting matter and spirit, nature and culture, subject and object, objectivity and subjectivity, science, and philosophy.

### Study limitations

Recommendations for conducting and subsequently reporting qualitative research were followed. However, assessing the contribution of Reiki at a single point in time following its application may represent a limitation of the findings.

### Contributions to the field of nursing and health

The scientific evidence presented in this study contributes to the field of nursing and health by reaffirming Reiki as a tool that enhances complex care and can and should be incorporated into the daily practice of professionals. Furthermore, it contributes to science by demonstrating the positive outcomes of Reiki application through the lens of the Complexity framework, serving as a reference for future research in the field.

## FINAL CONSIDERATIONS

This study made it possible to analyze the contribution of Reiki as a tool for promoting complex care. The main findings include changes in nurses’ feelings, self-knowledge, and self-awareness, which were reflected in altered behaviors and attitudes when facing daily uncertainties and in new ways of (co)existing and dealing with the processes of order, disorder, and reorganization. These changes led to healthier eating habits, improved sleep quality, increased energy and motivation for daily activities, and the adoption of regular physical exercise. Therefore, Reiki is shown to be a tool that enhances complex care and supports nurses in coping with the disorders experienced in their daily lives.

## Data Availability

The research data are available within the article.
