# Supplementary material for: Reiki as a tool to enhance care from the perspective of complexity: a qualitative study
Source: Rev Bras Enferm. 2025 Nov 7;78(4):e20250082. doi: 10.1590/0034-7167-2025-0082 (PMC12594479; doi:10.1590/0034-7167-2025-0082)
Supplement: Translation

# Translation: Reiki como ferramenta potencializadora de cuidado na perspectiva da complexidade: pesquisa qualitativa

**DOI:** 10.1590/0034-7167-2025-0082pt

**Authors:** Silvana Possani Medeiros, Adriane Maria Netto de Oliveira, Mara Regina Santos da Silva, Andreia Martins do Couto, Vagner Ferreira do Nascimento, Silomar Ilha

**Affiliations:** I Universidade Federal do Rio Grande. Rio Grande, Rio Grande do Sul, Brasil; II Espaço Terapêutico Andréia Martins do Couto. Rio Grande, Rio Grande do Sul, Brasil; III Universidade do Estado de Mato Grosso. Cáceres, Mato Grosso, Brasil; IV Universidade Federal de Santa Maria. Palmeira das Missões, Rio Grane do Sul, Brasil

**Keywords:** Toque Terapêutico, Dinâmica não Linear, Pesquisa Qualitativa, Cuidados de Enfermagem, Enfermagem.

## Abstract

**Objetivos:**

descrever as contribuições da aplicação do Reiki como ferramenta potencializadora de cuidado.

**Métodos:**

pesquisa exploratória, descritiva, qualitativa, com aplicação do referencial teórico-conceitual da complexidade, desenvolvida com dez enfermeiros de uma Unidade Básica de Saúde 24h no Sul do Brasil. Os dados foram coletados entre abril e agosto de 2022, por meio de entrevista semiestruturada individual, após cada participante receber cinco sessões de Reiki. Aplicou-se análise de conteúdo temática aos dados.

**Resultados:**

o Reiki contribuiu com alterações nos sentimentos, no autoconhecimento e autoconsciência dos enfermeiros, refletidas em alteração de comportamento/postura no enfrentamento das incertezas cotidianas e novas formas de (con)viver e lidar com processos de ordem-desordem-reorganização. Isso resultou em alimentação adequada, qualidade do sono, disposição e energia para realizar ações cotidianas, e prática de atividades físicas regulares.

**Conclusões:**

o Reiki mostra-se como ferramenta potencializadora do cuidado complexo que auxilia no enfrentamento das desordens vivenciadas no cotidiano dos profissionais de saúde/enfermagem.

## INTRODUÇÃO

Diversos são os fatores que acometem a raça humana, bem como as formas de interpretação e enfrentamento dos problemas que emergem ao longo da vida. Entre os fatores possíveis, destaca-se o estresse como uma reação psicofisiológica a agentes intrínsecos ou extrínsecos que geram desordens representadas nos seres humanos por meio de disfunções biopsicossociais, incluindo aumento da frequência cardíaca e da pressão arterial sistêmica, sudorese, mialgias, ansiedade, fadiga, irritabilidade, insônia, distúrbios alimentares, diminuição da capacidade de concentração, e outros efeitos nocivos à saúde do ser humano, o que, mediante a intensificação dos estímulos, produz diminuição do rendimento geral na vida cotidiana e prejuízo na sua saúde^(1)^. Sendo assim, quando não acompanhado/tratado adequadamente, podem surgir alterações de toda ordem^(2)^.

Embora a situação do estresse possa ocorrer em qualquer local e momento da vida, percebe-se, no ambiente laboral, um cenário potencializador dessa situação, a depender da natureza do trabalho. Nesse sentido, destaca-se a atuação dos profissionais da área da saúde, em especial, dos enfermeiros, que possuem o seu *lócus* de atuação clínica e de gestão em meio a vários elementos e aspectos que podem desencadear situações geradoras de desordem. Assim, os enfermeiros necessitam de (re)organização constante das suas condutas em relação aos agentes estressores, para que possam manter seu equilíbrio biopsicossocial e espiritual. Tais agentes podem ser classificados como advindos do meio interno e/ou externo, representados, por exemplo, por sentimentos produzidos a partir das vivências do cotidiano profissional ou por ambientes ocupacionais insalubres^(3)^.

Diante desse contexto, estratégias precisam ser (re)construídas e inseridas no ambiente de trabalho. Assim, ressalta-se a importância do pensamento da complexidade, que sinaliza a necessidade de não mais isolar, desunir e justapor, propondo um pensamento unificador, articulado, atento e compreensivo^(4)^. Entende-se que, a partir deste, seja possível promover o cuidado complexo dos profissionais de saúde, com a compreensão das certezas e incertezas, do separável e do inseparável, sem simplificar ou reduzir as vivências desses profissionais.

Nesse sentido, o cuidar dos profissionais de enfermagem na perspectiva da complexidade deve considerar a circularidade das situações propositivas de ordem, desordem e organização presentes nas interações do ambiente de trabalho. Estudo desenvolvido na Bahia descreve que o cuidado complexo na enfermagem deve compreender a multidimensionalidade do ser humano, considerando, além dos aspectos físicos, as dimensões emocionais, sociais e espirituais que também o compõem^(5)^. Essa compreensão deve ser válida quando a proposição do cuidado é direcionada tanto às pessoas a serem cuidadas pelos profissionais quanto aos profissionais como objeto do cuidado.

Assim, o cuidado complexo utilizado nesta pesquisa se refere àquele que compreende as multidimensões do ser humano (paciente ou profissional), que interagem entre si, formando o todo. Esse processo é influenciado pelo modo de interação das diferentes dimensões. Dessa forma, no que tange aos profissionais enfermeiros e seu ambiente laboral, é necessário encontrar estratégias para potencializar/promover a harmonia e equilíbrio das múltiplas dimensões que os compõem.

Entre as estratégias possíveis para promover o cuidado desses profissionais, salienta-se o Reiki como uma prática terapêutica, originária da cultura japonesa, que utiliza a imposição das mãos para a canalização da energia vital, com a finalidade de estimular os mecanismos naturais de recuperação e manutenção da saúde^(6)^. Busca-se, com essa ferramenta, um cuidado complexo, no qual o terapeuta reikiano é um canal capaz de conduzir e fazer fluir a energia universal para outros seres, atuando em diferentes níveis e equilibrando harmoniosamente os chakras, ou seja, os pontos relacionados às principais glândulas do corpo, com o intuito de restabelecer sua homeostase. É por meio dos chakras que o corpo humano se conecta, capta e transforma a energia que permeia o ser humano, além de distribuí-la ao longo do corpo, contribuindo para os processos de saúde^(7)^.

Nesse sentido, o Reiki é uma Prática Integrativa e Complementar em Saúde (PICS) que aborda o cuidado multidimensional biopsicossocioespiritual, considerando o ser humano como único e integrado. Esses traços, por natureza, o caracterizam como um fenômeno promotor de cuidado complexo. Contudo, são necessárias pesquisas que reafirmem as contribuições do Reiki como uma ferramenta/prática potencializadora de cuidado complexo. A esse respeito, sinaliza-se que existem estudos utilizando-o como prática terapêutica em diferentes contextos^(8-11)^. Entretanto, propõe-se, nesta pesquisa, uma discussão acerca do seu uso a partir do referencial teórico da complexidade, mais especificadamente a partir dos conceitos de ordem, desordem e (re)organização^(4)^, fato que justifica a relevância desta pesquisa.

Além disso, esta temática vincula-se ao terceiro objetivo “saúde e bem-estar” para o desenvolvimento sustentável, apresentado na Agenda 2030 da Organização das Nações Unidas^(12)^, o que vem ao encontro das recomendações do documento “Estratégias da Organização Mundial da Saúde sobre Medicina Tradicional 2014-2023”, orientando e incentivando a utilização das práticas tradicionais como tratamento complementar amplo nos sistemas de saúde^(13)^. Sob esse prisma, questiona-se: quais as contribuições da aplicação do Reiki como ferramenta potencializadora de cuidado?

## OBJETIVOS

Descrever as contribuições da aplicação do Reiki como ferramenta potencializadora de cuidado.

## MÉTODOS

### Aspectos éticos

Pesquisa derivada do macroprojeto intitulado “*Promoção da saúde mental ao longo do desenvolvimento humano*”, aprovado pelo Comitê de Ética em Pesquisa da Universidade Federal do Rio Grande. Foram seguidas as recomendações da Resolução n^o^ 466/12^(14)^, a qual aprova as diretrizes e normas regulamentadoras de pesquisas envolvendo seres humanos. Os participantes assinaram o Termo de Consentimento Livre e Esclarecido (TCLE) em duas vias, uma ficando com o participante, e a outra, com os pesquisadores. Para garantir o anonimato dos participantes, estes foram identificados pela letra “E” (enfermeiro), acompanhada de um algarismo, conforme a ordem das entrevistas (ex.: E1, E2, E10).

### Tipo de estudo

Trata-se de pesquisa exploratória e descritiva com abordagem qualitativa. No que tange à observância dos critérios de rigor metodológico para redação da pesquisa, utilizou-se o *COnsolidated criteria for REporting Qualitative research* ^(15)^.

### Cenário do estudo

Pesquisa realizada em uma Unidade Básica de Saúde (UBS) 24 horas, localizada em uma cidade do interior do Rio Grande do Sul, Brasil. A referida UBS, além de atender a comunidade local, presta serviços também à população de outros bairros em caráter de urgência e emergência, e em intervenções de maior complexidade. Além disso, atende pacientes que se encontram na situação de incompatibilidade de horário para se consultarem na UBS do seu bairro^(16)^.

### População

No período investigado, atuavam no cenário 36 profissionais assistenciais: dez enfermeiros, 16 técnicos de enfermagem, nove médicos e um cirurgião-dentista. Foram considerados os enfermeiros presentes no período de coleta de dados e que preencheram os critérios de seleção. A escolha pelos enfermeiros, para esta pesquisa, decorreu de serem os profissionais com alta demanda de trabalho, uma vez que, além da assistência direta aos pacientes, são responsáveis pela gestão do serviço.

### Critérios de seleção de participantes

Como critério de inclusão, considerou-se ser enfermeiro atuante na UBS 24 horas. Como critério de exclusão, consideraram-se profissionais que estivessem afastados do serviço por licença de saúde ou interesse no período da coleta dos dados. Todos os enfermeiros lotados na unidade, cenário da pesquisa, aceitaram participar do estudo e atenderam ao critério de inclusão, totalizando dez profissionais.

### Coleta de dados

Os dados foram coletados entre abril e agosto de 2022 em seis encontros com cada participante. No primeiro encontro, houve a apresentação da pesquisa aos enfermeiros, o aceite e a assinatura do TCLE. Do segundo ao sexto encontro, foram realizadas, uma vez por semana, sessões de Reiki, de forma individual, com cada um dos participantes. As sessões de Reiki foram realizadas por um dos pesquisadores (enfermeira, terapeuta reikiniana, com os três níveis de habilitação na técnica - Reiki níveis I, II, III). Ocorreram na UBS 24 horas, em um consultório disponível no momento da coleta, e o horário das sessões deu-se a partir da disponibilidade de cada participante.

Cada sessão de Reiki teve uma duração de 20 minutos e ocorreu da seguinte forma: a sala foi previamente preparada antes de cada encontro, com música relaxante, difusor de aromas, com intenção de proporcionar maior conforto para a realização das sessões, assim como harmonizar a sala. O participante foi convidado a deitar-se em uma maca em decúbito dorsal, sendo estimulado verbalmente pela pesquisadora a fechar os olhos, projetar imagens e afirmações positivas em seus pensamentos durante o recebimento do Reiki, de modo a ligar-se mais profundamente à prática.

Na sequência, a pesquisadora concentrava-se por alguns instantes e iniciava a aplicação de Reiki, com as palmas das mãos viradas para baixo, em forma de concha. Os primeiros chakras a serem harmonizados foram o coronário e o frontal, correspondendo às regiões frontais, temporais e occipitais da cabeça; logo depois, o chakra laríngeo, localizado nas regiões supra-hioidea e infra-hioidea; o chakra cardíaco, na região esternal; o chakra do plexo solar, situado na região abdominal do epigástrio; o chakra umbilical, localizado na região abdominal do mesogástrio; e o chakra base, situado na região púbica. Ainda na posição de decúbito dorsal, acessavam-se o chakra secundário do joelho, na região patelar, e o chakra secundário dos pés, na região plantar. Posteriormente, com o participante já na posição ventral, atuava-se no chakra cardíaco, na região escapular, no chakra do plexo solar, situado na região dorsal, e encerrava-se a sessão no chakra umbilical, na região lombar.

O sexto encontro ocorreu uma semana após a realização da última sessão de Reiki com cada participante. Nesse encontro, a pesquisadora, previamente capacitada e com experiência na técnica de coleta de dados, fez uma entrevista individual com cada participante no mesmo consultório em que foram realizadas as sessões de Reiki. As entrevistas tiveram duração média de 30 minutos e foram norteadas por um roteiro semiestruturado, construído especialmente para esta pesquisa, o qual era composto de duas partes. A primeira parte incluía a descrição dos participantes, como sexo, idade, tempo de formação e de atuação na unidade, renda, consumo de medicamentos, bebida alcóolica, prática de atividades físicas, lazer, e religião. A segunda parte incluía as seguintes questões abertas: fala-me sobre como você se sentia antes das sessões de Reiki. O Reiki influenciou na sua vida cotidiana? De que maneira? Você percebeu modificações na sua vida após a realização do Reiki? Quais?

As entrevistas foram audiogravadas por um gravador de voz e transcritas na íntegra, mecanograficamente, pelos pesquisadores, com o programa *Microsoft Word^®^* (versão 16.31).

### Tratamento e análise dos dados

Os dados foram analisados por meio da técnica de análise de conteúdo temática^(17)^, a partir da sequência: 1) Pré-análise, na qual foi realizada a leitura flutuante do material; 2) Exploração do material, situação na qual ocorreu a leitura profunda dos discursos advindos das entrevistas, em busca de afirmações que viessem elucidar acerca da contribuição do Reiki como uma ferramenta potencializadora de cuidado complexo e se ocorreram mudanças no cotidiano a partir da aplicação do Reiki, momento em que foram extraídas unidades de significado e com o seu agrupamento em categorias; e 3) Tratamento dos resultados obtidos e a interpretação à luz do referencial da complexidade^(4)^.

## RESULTADOS

Dos dez enfermeiros participantes da pesquisa, sete eram mulheres, e três, homens, com média de idade de 35 anos, com tempo médio de profissão de nove anos e tempo médio de atuação na unidade de um ano e oito meses. Quanto à renda, recebiam entre três e quatro salários mínimos. Cinco participantes referiram morar apenas com os filhos, e cinco, com o(a) companheiro(a). Quanto aos hábitos de vida, seis relataram fazer uso de bebida alcóolica pelo menos uma vez na semana; três faziam uso de medicação controlada (ansiolíticos); e cinco praticavam atividades físicas. Quanto às atividades de lazer, a maior prevalência observada foi de ver séries e filmes (n = 7). Cinco profissionais referiram seguir alguma religião, com a predominância do catolicismo (n = 3). Os dados analisados resultaram em três categorias, conforme a Figura 1.

**Figura 1:** Representação esquemática das categorias com base no referencial da complexidade

### Desordem pessoal gerada pelo estresse cotidiano: sentimentos e comportamentos

Compreende-se que o estresse é vivenciado de maneira singular pelos seres humanos, de acordo com suas próprias convicções e experiências. No entanto, havia aspectos semelhantes que ocorriam no cotidiano de trabalho dos enfermeiros participantes desta pesquisa, como a desordem pessoal ocasionada pelo excessivo estresse. Nas falas de dois participantes, é possível observar que o estresse cotidiano e as vivências do desconhecido, neste caso, a situação da COVID-19 e as incertezas geradas por ela, conduziram às alterações do humor, irritabilidade e impaciência.

*Claro que eu me estresso. Por exemplo, hoje eu estou uma pilha de nervos, tensa, porque, além dessa COVID-19, eu tenho que fazer vários pedidos, enviar relatórios, enfim, exercer toda a parte administrativa e de gestão da unidade para que tenham os recursos materiais mínimos necessários. Tudo junto é bem estressante! Me deixa bem irritada isso tudo!* (E1)

*Eu sou uma pessoa que não sou muito de levar desaforo para casa, assim, qualquer coisinha que me falavam. Eu já brigava, de repente eu até nem tinha razão, mas, se eu achasse que não, eu já me estressava. Ainda mais com essa COVID-19, a gente fica mais nervosa e com os nervos à flor da pele.* (E4)

Pode-se perceber que os sentimentos produzidos a partir das desordens vivenciadas pelo estresse no ambiente de trabalho tiveram reflexos tanto no cotidiano laboral quanto em aspectos da vida pessoal em família.

*Eu estava assim, com bastante problema em casa, irritado com meu filho, eu andava brigando muito, xingando mesmo. Ainda, para piorar, o meu colega de plantão, o outro enfermeiro, nós dois temos a personalidade forte, a gente tinha algumas discussões e que não eram legais. Eu cheguei a pensar em solicitar uma troca de turno ou dia de plantão, porque estava sendo muito difícil e estressante trabalhar com ele.* (E7)

*Eu não tinha nenhuma paciência. Algumas pessoas chegavam perto de mim e me faziam bastante mal, sentia um peso, quase um sofrimento de ter que aturar, principalmente aqui no trabalho, porque tem umas pessoas que são bem difíceis, não só os colegas, como alguns pacientes também.* (E10)

Os relatos dos enfermeiros mostram a desordem causada pela condição de estresse ou falta de compreensão a respeito de si mesmo no que se refere à consciência do eu e à clareza das suas emoções, o que, de certa forma, dificultava sua percepção a respeito do que realmente estava afetando o seu dia a dia. Assim, para além de alterações dos sentimentos, os problemas laborais do cotidiano influenciaram os comportamentos em desacordo com a manutenção da saúde, como a qualidade do sono, alimentação inadequada e desânimo no desempenho para as atividades no âmbito do trabalho.

*Eu tinha o sono muito agitado, demorava para dormir. Tinha dias que parecia que nem tinha dormido, porque acordava mais cansada do que quando deitei* [...] *percebo a minha ansiedade e compulsão alimentar pela quantidade de comida que eu comia. Eu precisava comer o tempo todo e comida de péssima qualidade; muitas vezes só um fast food me satisfazia.* (E3)

*Meu sono tem estado meio ruim, sabe? Porque parece que eu estou sempre cansado, com um peso no corpo, deve ser por causa da tensão em relação a esta doença, a COVID-19, que não acaba nunca. Fora isso, os afazeres diários aqui no trabalho são tensos, estressantes, saio esgotado.* (E5)

*Eu tenho me alimentado mal, porque eu sou dessas que desconta minha ansiedade na comida, tenho comido muitos doces e fast food* [...] *geralmente, quando chegava em casa depois do trabalho, me sentia esgotada e não tinha vontade nenhuma de sair de casa, nem de ver ninguém, só de dormir.* (E9)

Os relatos revelam a desordem decorrente do estresse no cotidiano dos enfermeiros. A desorganização de suas rotinas, tanto na esfera familiar quanto na esfera do ambiente de trabalho, gerou sensações de irritabilidade, nervosismo e cansaço, levando-os a comportamentos de descuidado.

### Ordem pessoal a partir do Reiki: mudança no sentir, pensar e gerenciar o cotidiano

A desordem manifestada a partir dos sentimentos de desânimo, sobrecarga e impaciência, e da instabilidade do humor, aos poucos, passou a ser reinterpretada a partir do Reiki, resultando em uma nova ordem, ou seja, novas maneiras de enxergar, (con)viver e gerenciar as atividades do cotidiano. Após a intervenção reikiana, alguns enfermeiros adotaram uma postura diferente, adquiriram outra consciência sobre si e passaram a lidar melhor com situações que antes lhes eram desconhecidas, de acordo com os relatos a seguir:

*Eu, agora, depois de ter recebido o Reiki estou pensando assim: “Ah, eu tenho que fazer tal coisa. E se não conseguir, não consegui, não vou me torturar e sofrer por causa disso”. Entende? Eu tenho respirado muito mais, tentado me acalmar, refletido mais.* (E1)

*Ah, é como se eu conseguisse lidar melhor com as adversidades do dia a dia. Tive um pouco mais de equilíbrio emocional para resolver problemas que a gente vivencia em casa e no trabalho, na vida de modo geral. Estou mais tranquila depois que recebi o Reiki.* (E2)

*Eu percebo que agora eu penso mais em mim, eu respiro mais devagar, penso com mais clareza, fazendo as coisas com mais calma e tranquilidade, focando no que realmente é importante para mim. Muito obrigada pelo Reiki, realmente me ajudou.* (E8)

*Agora, depois de receber o Reiki, eu tento pensar mais nas coisas pelo lado positivo, tento filtrar o que eu estou ouvindo, se a pessoa está perto de mim falando, eu seleciono o que é importante para eu ouvir, se aquilo vai acrescentar em algo na minha vida ou não, isso eu estou mudando e me ajuda muito*. (E10)

Nota-se nos discursos uma mudança no pensamento e, a partir disso, no comportamento dos enfermeiros diante das incertezas do dia a dia, após a intervenção reikiana. Esses profissionais mencionaram a nova ordem instituída, a partir das transformações sentidas e da dinâmica que se estabeleceu em suas rotinas, visto que iniciaram um processo de reestruturação, tanto interna, a partir de uma nova ordem de pensamento, quanto externa, com nova ordem de comportamento.

### (Re)organização a partir do Reiki: mudanças de comportamento no cotidiano

As experiências compartilhadas pelos enfermeiros demonstram que, após a intervenção reikiana, além de uma nova ordem, por meio da mudança na forma de pensar e de sentir, ocorreu o processo de (re)organização, demonstrado a partir da mudança de comportamento. Percebe-se um ciclo no qual a desordem resultou em uma nova ordem, levando a uma (re)organização do cotidiano.

*Depois do início das sessões de Reiki, eu estou me sentindo mais calma. Deu uma tranquilizada, reorganizei as coisas da minha vida, em geral. Eu não tenho me irritado* [...] *eu procurei me mexer de algum jeito, fazer alguma atividade física. Comecei a caminhar, não é todo dia, mas já me sinto mais disposta*. (E1)

*Agora estou me permitindo fazer outras coisas fora do trabalho. Às vezes, eu vou andar de bicicleta; às vezes, eu saio para dar uma caminhada e não fico o tempo todo só focada nas coisas que me estressam, nos problemas. Acho que estou me organizando melhor agora.* [...] *estou menos ansiosa até para comer, comendo menos e mais equilibrada, comendo fruta que antes eu não comia, comendo mais devagar, bebendo mais água, porque o trabalho vai estar lá igual quando eu voltar do intervalo.* (E3)

*Eu sentia dor nos meus pés por ficar 12 horas praticamente em pé aqui no posto e dor na lombar, daí tu fez o Reiki e passou, assim na hora. Principalmente no dia que era feito, mas diminuiu de modo geral, me mantive sem dor* [...] *depois do Reiki, durmo melhor, sem interrupções, com mais qualidade, acordo menos cansada. Isso acaba dando mais disposição durante o dia, tenho mais energia e ânimo para fazer as coisas.* (E4)

*Eu percebi que a relação com meus pais melhorou, e eles dizem que eu fiquei mais tranquilo, que eu comecei a falar com mais calma, mais paciência, depois das sessões do Reiki* [...] *percebo que tenho mais disposição agora, voltei até a malhar, fazer musculação. Tenho mais ânimo até para estudar.* (E6)

Nota-se, por meio dos relatos dos enfermeiros, que foi possível (re)organizar-se com vistas ao gerenciamento do estresse cotidiano, por meio da utilização do Reiki como ferramenta de cuidado complexo. (Re)organizaram-se algumas emoções e comportamentos, ocasionando uma nova ordem manifestada pela mudança do que estava conduzindo à desordem.

## DISCUSSÃO

A complexidade do ser enfermeiro abarca aspectos relacionados ao cuidado diante das diversas situações, considerando a complexidade do ser humano; aos desafios nos relacionamentos interpessoais e interprofissionais; à relação do ser humano/pessoa cuidada pelo enfermeiro; à terminalidade da vida; e ao aprofundamento do conhecimento relativo à complexidade do cuidado no âmbito da integralidade da assistência^(18)^. Nesse sentido, os enfermeiros podem vivenciar situações de estresse por trabalharem diretamente na assistência e cuidados, vivenciando, por vezes, momentos de incertezas, medos e sentimento de impotência diante do sofrimento e morte.

Na presente pesquisa, revelou-se a desordem produzida pelo estresse na vida dos enfermeiros, a qual repercutiu na desorganização de suas rotinas, alterando o convívio na esfera familiar e no ambiente de trabalho. Tal desordem foi manifestada a partir de sentimentos como irritabilidade, nervosismo e cansaço, os quais se intensificaram pela situação da *coronavirus disease* (COVID-19). Esse dado corrobora revisão de literatura que buscou a repercussão na saúde mental do enfermeiro no período da pandemia de COVID-19. O referido estudo demonstrou que os profissionais de saúde lidaram com emoções e sentimentos no âmbito laboral e passaram por altos níveis de estresse e tensão, os quais repercutiram no maior risco para o desencadeamento de irritabilidade, apatia, desânimo, angústia, ansiedade, entre outros sentimentos^(19)^.

A esse respeito, cabe mencionar que algumas emergências de saúde pública, como a produzida pela COVID-19, por exemplo, suscitam o aumento das taxas de ansiedade, depressão e outros transtornos mentais nos profissionais, além de promoverem comportamentos sociais negativos, como irritabilidade e impaciência, o que pode implicar diretamente a sua saúde biopsicossocial, assim como a eficácia da sua atividade laboral^(20)^. Para além da desordem relacionada às sensações e sentimentos, pode-se constatar que a exposição a altos níveis de estresse conduziu os enfermeiros participantes da presente pesquisa a comportamentos não saudáveis, como alimentação inadequada, desordem relacionada ao sono sem efeito reparador e cansaço após acordar, resultando em desânimo para o trabalho. Destaca-se que é comum, em situações de estresse, a manifestação de comportamentos alimentares inadequados, justificando-os como recompensa alimentar. Tal situação é caracterizada como comer na ausência de fome e a escolha por alimentos menos nutritivos e com alto teor de gordura e carboidratos. Assim, a desordem produzida pelo estresse ocasiona comportamentos alimentares desordenados associados comumente à compulsão alimentar^(21)^.

Estudo internacional, realizado com enfermeiros atuantes na assistência ao paciente com COVID-19, demonstrou dados semelhantes aos encontrados nesta pesquisa. No referido estudo, os profissionais foram acometidos por níveis elevados de estresse e sofreram impactos diretamente relacionados à qualidade do sono e repouso, pela duração insatisfatória, com presença de pesadelos, interrupções do sono, ou ao despertar pela manhã^(22)^.

Na presente pesquisa, pode-se perceber uma nova ordem produzida após as cinco sessões de Reiki, pois os enfermeiros passaram a compreender que os contratempos e adversidades não deixariam de acontecer no dia a dia e que seria impossível controlar tudo ao seu redor, pois conviviam constantemente com situações inesperadas. Alguns relatos permitiram inferir que era possível lidar de outro modo com as emoções e aspectos considerados negativos no cotidiano, a partir da compreensão de que possuíam pouco ou nenhum controle sobre os desafios cotidianos. No que concerne às relações interpessoais percebidas inicialmente como difíceis, tensas e estressantes, os resultados apontaram melhor aceitação das diferenças pelos participantes, ao perceberem que, apesar de algumas questões estarem fora de seu controle, manter a mente calma era melhor, havendo mudanças em seu interior, através da reflexão e ressignificação de atitudes e comportamentos.

Essa mudança de pensamento se deve ao fato de o Reiki promover o olhar do ser humano para si, para o seu eu interior, estimulando o autoconhecimento e a reflexão acerca do que está à sua volta, aumentando a sensação de segurança, o bem-estar e a manifestação de sentimentos positivos^(7)^. Destaca-se, dessa forma, que o Reiki como ferramenta de cuidado proporcionou a expansão de consciência, levando-os à autoanálise e compreensão mais aprofundada da vida. Assim, os enfermeiros passaram a ter outra percepção a respeito dos acontecimentos experienciados, proporcionando um melhor discernimento e enfrentamento das adversidades do cotidiano.

Nesse sentido, o Reiki demonstrou sua contribuição como ferramenta potencializadora de cuidado complexo, uma vez que promoveu uma nova ordem de pensamento e sentimento que possibilitou a (re)organização de comportamentos relacionados à alimentação, contribuindo para uma escolha mais equilibrada dos alimentos. Dessa forma, os resultados da presente pesquisa reafirmam o apresentado na Política Nacional de Práticas Integrativas e Complementares, que apresenta o Reiki como uma prática que promove a articulação das dimensões psíquica, emocional, física e espiritual, compreendendo a singularidade e multidimensionalidade do ser humano^(6)^, vindo de encontro às ideias do modelo médico-centrado que fragmenta o ser humano em partes isoladas, sem necessariamente contextualizar o todo.

Após o Reiki, houve relatos sobre melhora na qualidade do sono, maior disposição e energia para realizar ações cotidianas, incluindo o início de atividades físicas regulares. Esse dado corrobora os de estudo com enfermeiros, o qual evidenciou os impactos positivos do Reiki na qualidade do sono e na diminuição das dores físicas, as quais foram amenizadas ou eliminadas^(23)^. Outras pesquisas desenvolvidas com vistas a conhecer as repercussões do Reiki no cotidiano dos enfermeiros encontraram resultados semelhantes aos da presente pesquisa, pois identificaram melhoria na qualidade do sono, redução das dores e do cansaço diurno, melhora da disposição para as atividades diárias^(24,25)^, aumento da autoestima, e expansão da consciência sobre si, sobre a família, sobre o trabalho e sobre a vida em geral^(25)^.

Tais resultados foram possíveis, haja vista que o Reiki realiza desbloqueios energéticos, e o ser humano começa a se perceber mais tranquilo, devido ao relaxamento da musculatura corporal, assim como estimula a produção de serotonina e endorfina, hormônios que promovem o bem-estar e o relaxamento. Dessa forma, harmoniza e equilibra o corpo que, consequentemente, entra em sintonia e busca por alimentos mais saudáveis, assim como hidratar-se, o que ajuda a potencializar o efeito desintoxicante do Reiki e a estimular seus benefícios^(7)^. No que se refere ao aumento da sensação de bem-estar e da disposição, ele ocorre devido à canalização de energia de forma intencional, no intuito de restaurar o equilíbrio do campo energético que todo o ser vivo possui, o que, consequentemente, reflete na melhora de bem-estar em todas as suas dimensões^(7)^.

Esse resultado positivo é esperado, pois o Reiki é um recurso para a promoção do equilíbrio dos chakras, refletindo nas glândulas endócrinas; atuando na homeostase do sistema nervoso; dissipando traumas emocionais; liberando emoções estagnadas; promovendo a expansão da consciência; despertando a pessoa para a conexão com a Energia Universal; expandindo a conexão do ser humano com o seu eu superior; e aumentando a sensibilidade, a criatividade e a intuição.

Assim, é possível perceber que o Reiki produziu impactos positivos no cotidiano dos profissionais, evidenciados pela (re)organização nos diferentes aspectos das suas vidas. Esse dado corrobora os de pesquisa desenvolvida nos Estados Unidos, onde os participantes foram solicitados a relatar suas vivências durante as sessões de Reiki, por meio de um questionário logo após o término das sessões. Os pesquisadores obtiveram um total de 1.284 respostas, as quais foram agrupadas em oito categorias principais: aumento do relaxamento e tranquilidade profunda (68%); ampliação de sensações corporais/experiências somáticas positivas (53%); melhora da regulação e controle emocional (29%); significado espiritual ou simbólico (18%); melhora nos sintomas físicos relacionados às dores (17%); estados reflexivos e alterações na percepção (11%); indução ao sono e sonolência (10%); e estabilização da respiração (4%)^(26)^.

Outra pesquisa, realizada pela Universidade Estadual do Rio de Janeiro, que objetivou reduzir a carga de estresse do profissional de saúde através do cuidado integral fazendo uso de PICS, como auriculoterapia, Reiki, reflexologia podal e massagens, demonstrou resultado positivo. Nesse sentido, as PICS mostraram-se como estratégias efetivas e viáveis de atenção ao trabalhador da saúde, pois, ao incorporá-las neste cenário, aproximaram-se os saberes, articularam-se práticas e vivências, promoveram-se a saúde mental e a qualidade de vida por meio de intervenções de um cuidado efetivo^(27)^.

Cabe mencionar que as PICS não possuem o intuito de isolar e separar, mas, sim, estabelecer a boa convivência entre racionalidades complementares e práticas que dispõem de outras configurações de um cuidado mais abrangente, refletindo no bem-estar e, concomitantemente, na assistência prestada desses trabalhadores^(27)^. Nesse sentido, os significados e as experiências com o Reiki são inúmeros, mas convergem para a compreensão dessa prática como produtora de saúde, bem-estar e qualidade de vida, por meio do cuidado multidimensional ao ser humano^(28)^.

Nessa perspectiva, reconhece-se a complexidade dos processos de mudança, das relações entre diferentes níveis e dimensões de um mesmo fenômeno. A partir desse paradigma, o cuidar envolve sempre o outro que recebe o cuidado, constituindo, assim, uma via de mão dupla, em que há necessidade de criar vínculos para a efetividade do ato de ajudar o outro a ser protagonista e promotor da sua saúde. O Reiki, assim como as demais PICS, vem ao encontro deste pressuposto, pois estabelece naturalmente a criação e o fortalecimento de vínculos, promovendo um cuidado multidimensional, ou seja, em que se considera o ser humano na sua singularidade e a influência da sua individualidade em relação aos outros e em diferentes contextos, assim como esses também interferem na saúde e qualidade de vida de cada ser humano^(29)^.

O referencial da complexidade mostra que o ser humano não deve se limitar a um único campo de conhecimento, uma vez que a unidade humana se constitui na multidimensionalidade e o pensamento complexo põe em evidência outros modos de usar a lógica^(5)^. A partir dessa perspectiva, é necessário compreender o cuidado de enfermagem como uma prática social e dinâmica que reconhece as igualdades e diferenças biológicas, sociais, culturais e políticas, reconhecendo a complexidade da inter-relação do ser humano com as relações do processo de cuidado.

Por isso, o Reiki como ferramenta de cuidado complexo surge como uma forma de cuidado com os profissionais de saúde/enfermagem que repercutirá na assistência desenvolvida pelos mesmos, pela óptica de uma práxis inovadora, com possibilidade de renovação, considerando todas as experiências de que a consciência dispõe. Portanto, a complexidade e o Reiki estabelecem uma estreita relação, em que ambos congregam e articulam os diversos saberes que compõem os mais variados campos do conhecimento, sem perder a essência e o fio condutor das singularidades, ou seja, é religar matéria, espírito, natureza e cultura, sujeito e objeto, objetividade e subjetividade, e ciência e filosofia.

### Limitações do estudo

Foram seguidas as recomendações para a condução e posterior relatório de pesquisas qualitativas. Contudo, ter avaliado a contribuição do Reiki, em um único momento após a sua aplicação, pode se constituir como uma limitação dos achados.

### Contribuições para as áreas da enfermagem e saúde

As evidências científicas obtidas contribuem para a enfermagem/saúde, uma vez que reafirmam o Reiki como uma ferramenta potencializadora de cuidado complexo, o qual pode/deve ser empregado no cotidiano laboral dos profissionais. Além disso, ressalta-se a contribuição para a ciência, pois demonstram os resultados positivos da aplicação do Reiki, a partir do referencial da complexidade, podendo servir como referência para outras pesquisas na área.

## CONSIDERAÇÕES FINAIS

Esta pesquisa possibilitou analisar a contribuição do Reiki como ferramenta promotora de cuidado complexo. Como principais contribuições, destacaram-se mudanças de sentimentos dos enfermeiros, no autoconhecimento e autoconsciência, refletidas na mudança de comportamento e postura no enfrentamento das incertezas do cotidiano e novas formas de (con)viver e lidar com os processos de ordem-desordem-reorganização. Tais mudanças conduziram a uma alimentação adequada, melhora na qualidade do sono, maior disposição e energia para realizar ações cotidianas e a introdução da prática de atividades físicas regulares. Portanto, o Reiki mostra-se como ferramenta potencializadora do cuidado complexo que auxilia no enfrentamento das desordens vivenciadas no cotidiano dos enfermeiros.

## Data Availability

Os dados da pesquisa estão disponíveis no corpo do artigo.
